# Supplementary material for: Molecular Characterization of the MoxR AAA+ ATPase of Synechococcus sp. Strain NKBG15041c
Source: Int J Mol Sci. 2024 Sep 15;25(18):9955. doi: 10.3390/ijms25189955 (PMC11432383; doi:10.3390/ijms25189955)
Supplement: Supplementary file 1 [file ijms-25-09955-s001.zip › ijms-3185483-supplementary.pdf]

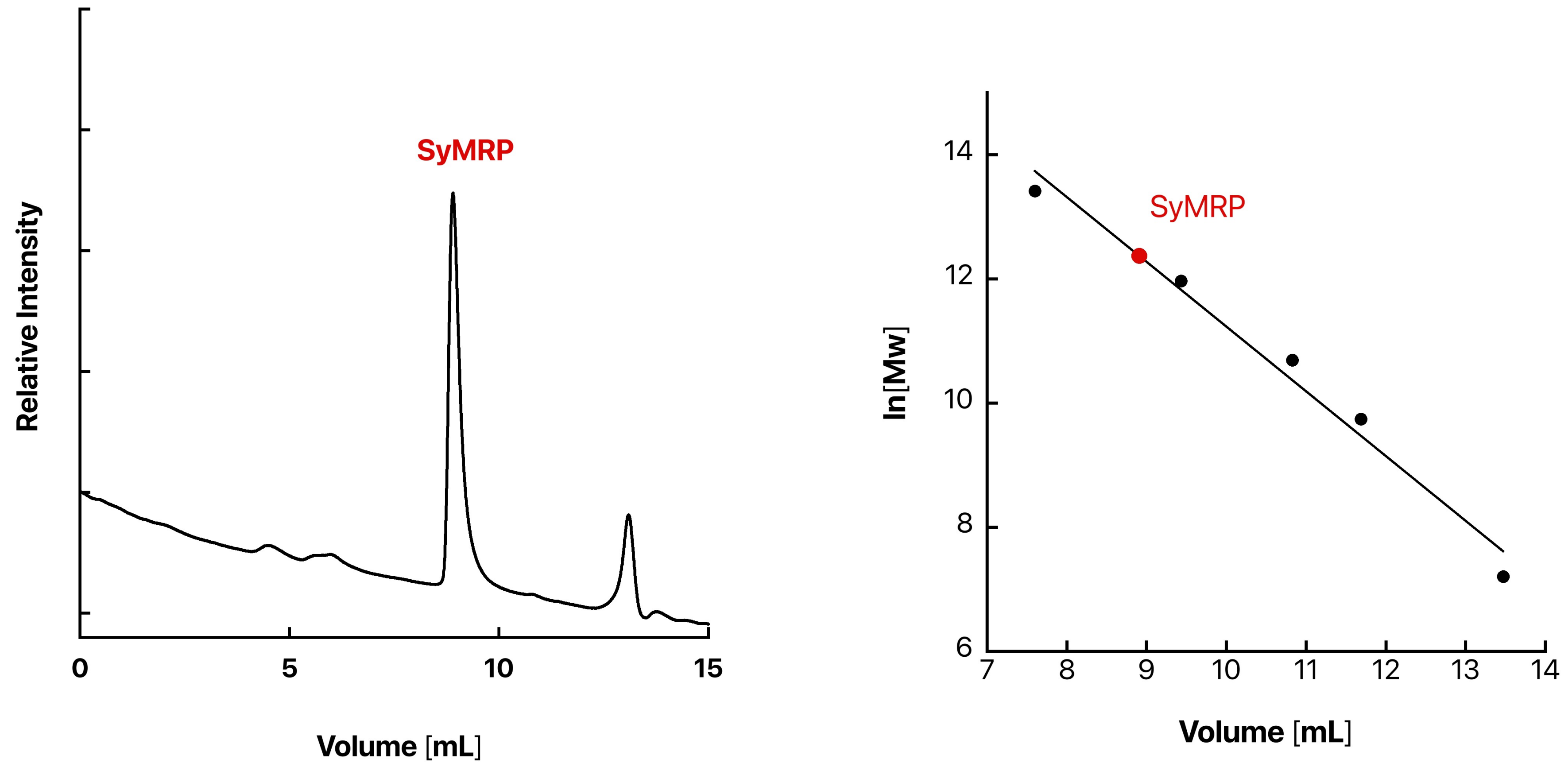

Supplementary Figure S1 Size exclusion chromatography of SyMRP (Left) Chromatogram (Right) Estimation of Molecular Mass
